# Gene Sets and Mechanisms of Sulfate-Reducing Bacteria Biofilm Formation and Quorum Sensing With Impact on Corrosion

**DOI:** 10.3389/fmicb.2021.754140

**Published:** 2021-10-29

**Authors:** Abhilash Kumar Tripathi, Payal Thakur, Priya Saxena, Shailabh Rauniyar, Vinoj Gopalakrishnan, Ram Nageena Singh, Venkataramana Gadhamshetty, Etienne Z. Gnimpieba, Bharat K. Jasthi, Rajesh Kumar Sani

**Affiliations:** ^1^Department of Chemical and Biological Engineering, South Dakota School of Mines and Technology, Rapid City, SD, United States; ^2^2-Dimensional Materials for Biofilm Engineering, Science and Technology, South Dakota School of Mines and Technology, Rapid City, SD, United States; ^3^Data Driven Material Discovery Center for Bioengineering Innovation, South Dakota School of Mines and Technology, Rapid City, SD, United States; ^4^BuG ReMeDEE Consortium, South Dakota School of Mines and Technology, Rapid City, SD, United States; ^5^Department of Civil and Environmental Engineering, South Dakota School of Mines and Technology, Rapid City, SD, United States; ^6^Biomedical Engineering Program, University of South Dakota, Sioux Falls, SD, United States; ^7^Department of Materials and Metallurgical Engineering, South Dakota School of Mines and Technology, Rapid City, SD, United States; ^8^Composite and Nanocomposite Advanced Manufacturing Centre–Biomaterials, Rapid City, SD, United States

**Keywords:** sulfate reducing bacteria, biofilm, microbiologically influenced corrosion, comparative genomics, biocides, 2D-materials, quorum sensing, biocorrosion mechanism

## Abstract

Sulfate-reducing bacteria (SRB) have a unique ability to respire under anaerobic conditions using sulfate as a terminal electron acceptor, reducing it to hydrogen sulfide. SRB thrives in many natural environments (freshwater sediments and salty marshes), deep subsurface environments (oil wells and hydrothermal vents), and processing facilities in an industrial setting. Owing to their ability to alter the physicochemical properties of underlying metals, SRB can induce fouling, corrosion, and pipeline clogging challenges. Indigenous SRB causes oil souring and associated product loss and, subsequently, the abandonment of impacted oil wells. The sessile cells in biofilms are 1,000 times more resistant to biocides and induce 100-fold greater corrosion than their planktonic counterparts. To effectively combat the challenges posed by SRB, it is essential to understand their molecular mechanisms of biofilm formation and corrosion. Here, we examine the critical genes involved in biofilm formation and microbiologically influenced corrosion and categorize them into various functional categories. The current effort also discusses chemical and biological methods for controlling the SRB biofilms. Finally, we highlight the importance of surface engineering approaches for controlling biofilm formation on underlying metal surfaces.

## Introduction

Sulfur is an abundant element that exists in the forms of pyrite (FeS_2_), gypsum (CaSO_4_), and sulfate in the natural environments, including rocks, sediments, and seawater, respectively (Labrado et al., 2019). Some bacteria can use sulfur to generate amino acids (e.g., cysteine and methionine) and enzymes (Muyzer and Stams, 2008) required to generate metabolic energy. Sulfur cycling in the environment is dominated by sulfate-reducing bacteria (SRB) and sulfur-oxidizing bacteria (SOB) (Zhang et al., 2017). SRB gain energy through dissimilatory sulfate reduction, which is the main process of biomineralization of organic matter in marine sediments. SRB respire using sulfate as a terminal electron acceptor, reducing it to hydrogen sulfide. SRB are of primary concern because they accelerate metallic corrosion under mild conditions, including neutral pH, ambient temperature, and absence of oxygen, both in natural and engineered aquatic systems (Lee et al., 1995; Beech and Sunner, 2007). SRB are often responsible for microbiologically influenced corrosion (MIC), accelerating corrosion reactions, or shifting corrosion mechanisms (Venzlaff et al., 2013; Guan et al., 2016).

An essential step used by microorganisms to effectively induce MIC is to grow into a biofilm on underlying metal surfaces. Such biofilms allow them to grow even under hostile conditions and promote redox processes on metal surfaces responsible for accelerating metal corrosion (Procópio, 2019). The formation of biofilm structure and its maintenance are vital factors that influence the kinetics of the MIC process. Biofilms are stratified structures where the deeper layers in contact with the metal surface maintain anoxic conditions. These anoxic niches promote the growth of specific microorganisms, including SRB, that use the metal compounds as resources for their survival (Procópio, 2019). The mechanisms of biofilm formation by SRB involves a plethora of differentially regulated genes (Krumholz et al., 2015; Qi et al., 2016). In general, SRB biofilms are composed of proteins with a minimal exopolysaccharide content (Beech et al., 2005; Clark et al., 2007, 2012). Prior studies on other aerobic or facultative anaerobic microbes have explored differential gene expression and protein profiles in planktonic cells and in biofilms. These studies have revealed that biofilm formation is a complex and yet highly regulated process (Resch et al., 2006; Qi et al., 2016; Sánchez et al., 2019). This review discusses the transcriptional and proteomic variations in SRB biofilm and key genes involved in biofilm formation and subsequent corrosion effects.

While corrosion is a relatively simple process, assessing the relevant *in situ* and localized mechanism is challenging. Aggravating this problem is the presence of biofilm, which consists of microbial cells adhered to surfaces that influence the electrochemical reactions on the corroding surfaces. These cells release several metabolites and proteins that cause secondary effects on MIC (Beale David et al., 2016). To fully understand these effects, it is essential to analyze changes at the gene and protein level. Until now, most of the review articles have concentrated on electrochemical and thermodynamic aspects of MIC (Li et al., 2018; Lv and Du, 2018). Although the biological and electrochemical mechanisms of MIC in sulfate-rich environments have been extensively reviewed (Enning and Garrelfs, 2014; Gu et al., 2019), an extensive discussion on the molecular and regulatory mechanisms of biofilm formation by SRB is lacking. Biofilm build-up of SRB accelerates metal degradation by creating favorable conditions for microbes to corrode metals (Zhang et al., 2007; Procópio, 2019). The present review describes the process of SRB biofilm formation at the genetic and molecular levels. Noting that the underlying molecular mechanisms of adaptation are influenced by environmental factors (e.g., pH, temperature, and O_2_ and C levels) and surface characteristics (Toyofuku et al., 2016), we unveil such molecular mechanisms using omics data. We also discuss the underlying physiological adaptation between the biofilm and planktonic growth modes, which is scarce.

## Crucial Genes and Proteins in Biofilm Formation and Microbiologically Influenced Corrosion

A sulfate-reducing bacterium has genes for every activity and function and expresses them in corresponding proteins to form biofilms on different surfaces under various environments. These proteins later induce microbiological corrosion on the metal surfaces. Unlike the planktonic counterparts, the sessile cells in SRB biofilms use a unique combination of genes and proteins to establish a distinct flow of energy and cell associations (Price et al., 2014; De León et al., 2017). Studies based on targeted mutagenesis and gene expression analyses have shown an increased expression of genes coding for exopolysaccharides (EPS) (Clark et al., 2007; Qi et al., 2016). However, it has been reported that SRB biofilm is composed mainly of proteins with minimal EPSs (Beech et al., 2005). There have been very few studies available on genes and proteins on a molecular level in SRB other than *Desulfovibrio vulgaris* Hildenborough (DVH) and *Desulfovibrio alaskensis* G20 (DA-G20). DVH and DA-G20 have been used as model organisms for studying biofilm formation and MIC aspects of SRB. A large number of SRB studies have focused on these two species, and thus, information on genes responsible for biofilm formation has also been depicted using them (Clark et al., 2007; Elias et al., 2009; Price et al., 2014; Williamson et al., 2018). It has been found that biofilms of *D. vulgaris* showed altered levels of transcripts and proteins involved in critical functional categories such as carbon and energy metabolism, amino acid metabolism, stress response, proteases, and ribosomal proteins (Clark et al., 2012; Zhang et al., 2016; De León et al., 2017; Zhu et al., 2019).

Maximum studies on SRB biofilm formation used lactate as an electron donor and sulfate as an electron acceptor. Lactate–sulfate is the standard media used to study SRB biofilm characteristics (Clark et al., 2006; Brileya et al., 2014). However, the type of surfaces for biofilms (metal or non-metal) varies among different studies (Brileya et al., 2014; Dec et al., 2017; Krantz et al., 2019). Lopes et al. (2006) reported that metal (stainless steel) composition plays a significant role in the development of the biofilm of DA G20. They demonstrated that high nickel concentration had a negative effect on cell growth, while low concentration showed a positive impact. In contrast, chromium on the metal surface did not affect cell duplication. Chen et al. (1998) also reported growth retardation of DA G20 in the presence of molybdenum. Previous studies have shown that biofilm cells grown in lactate/sulfate media have different expression profiles than planktonic cells grown under the similar conditions (Clark et al., 2012; Sivakumar et al., 2019).

A transcriptomic study showed that a total of 358 genes downregulated in biofilms as compared to planktonic cells (Clark et al., 2012). Out of these 358 genes, 10% have been predicted to be involved with translation and ribosomal structure/biogenesis. Downexpression was also observed in genes for amino acid transport and metabolism, cell synthesis, and cell division. Besides the down-regulated genes, a higher expression was recorded for the number of gene including hydrogenase and formate dehydrogenases and in genes involved in energy production and conservation, signal transduction, and cell motility in SRB biofilms as compared to planktonic cells. A 26-fold higher rate of formate-dependent methyl viologen reduction was reported in the biofilm, indicating increased gene expression and enzymatic activity. The critical genes in SRB (DA-G20) that have been reported to be essential for biofilm formation are given in Table 1.

**TABLE 1:** Selected genes that impact biofilm formation by sulfate-reducing bacteria (SRB).

**Gene Id**	**SRB**	**Annotation**	**Role in Biofilm formation**	**References**
DVU1017	DVH	ABC transporter of a type I secretion system	Localization of adhesion proteins on the cell surface	De León et al., 2017
DVU1012	DVH	Hemolysin-type calcium-binding repeat-containing protein	Cell communication and adhesion *via* plasma membrane adhesion molecules^a^	De León et al., 2017
DVU0281	DVH	Exopolysaccharide biosynthesis protein	Biofilm formation and metabolism	Ren et al., 2004; Qi et al., 2016
Dde_2358^b^	DAG	Pilus assembly protein	Cell adhesion^a^	Krumholz et al., 2015
DVU1340	DVH	Ferric uptake repressor protein	Maintaining normal metabolism in DVH biofilm under high concentration of iron	Qi et al., 2016
Dde_0430^b^	DAG	Cell wall biogenesis glycosyltransferase-like protein	Modulates dynamic interactions between glucan and extracellular DNA (biofilm matrix components)	Krumholz et al., 2015; Rainey et al., 2019
Dde_3584^b^	DAG	Flagellar biosynthesis protein	Powers swimming through liquid and swarming over solid surfaces	Kearns, 2010; Krumholz et al., 2015
DVU1817	DVH	Cytochrome c553	Electron transfer partner for the formate dehydrogenase in DVH biofilms	Clark et al., 2012
DVU0752	DVH	ABC transporter	Translocation of a wide variety of molecules into or out of biofilm cells	Shemesh et al., 2007; Clark et al., 2012
DVU0330	DVH	Response regulator containing a metal-dependent phosphohydrolase	Metal stress tolerance in biofilm cells	Rajeev et al., 2014

*DVH, *Desulfovibrio vulgaris* Hildenborough; DAG, *Desulfovibrio alaskensis* G20.*

*^*a*^Function was verified from UniProt protein database.*

*^*b*^Gene Id obtained from STRING database.*

### Functional Categories of Genes and Proteins Involved in Sulfate-Reducing Bacteria Biofilm Formation

Sulfate-reducing bacteria biofilm have been reported to have altered expression of transcripts and proteins at a different level of growth and nutrient availability when compared with planktonic cells (Clark et al., 2012; Krumholz et al., 2015; Qi et al., 2016). It is considered that biofilms have different levels of expression of genes and their corresponding proteins. To sustain the unique state of biofilm growth, the primary requirement is energy generation, and therefore, the expression of genes required for energy conversion and carbon flow is one of the significant activities of the biofilm. It has been found that biofilm cells have differences in the expression of genes of two pyruvate:ferredoxin oxidoreductases [e.g., *oor* (upregulated) and *por* (downregulated)] as compared to planktonic cells. Although the expression of pyruvate formate lyase was unchanged, formate dehydrogenase expression (hybA transcript and protein, alpha subunit) was found to increase. Only protein abundance was found to increase for fdnG1 (beta subunit of formate dehydrogenase). At the same time, cytochrome c553 (electron transfer partner for formate dehydrogenase) was found to increase for both mRNA and protein (Sebban-Kreuzer et al., 1998) in DVH. It has also been reported that SRB biofilm has 26-fold higher formate-dependent (dehydrogenase) activity than planktonic cells and assumed that it is because of formate cycling and their role in electron flow (Voordouw, 2002; Heidelberg et al., 2004; Clark et al., 2012). Some other dehydrogenases were also reported in the biofilm, and their roles in proton and electron cycling need to be explored to understand the mechanisms in different states of SRB biofilm.

#### Genes and Proteins Involved in Carbohydrate and Nitrogen Metabolism

Carbon uptake and utilization is one of the major requirements to drive energy and biosynthesis of several essential molecules and compounds for bacteria to survive. Previous studies have reported that SRB have several genes, which give flexibility to uptake and utilize different carbon compounds like glycerol and phospholipids and could be processed for amino acid biosynthesis. The observation was supported by the downexpression of glycolytic pathway and gluconeogenesis required for amino acid biosynthesis and amino-sugar biosynthesis, respectively followed by decreased expression of genes responsible for cell wall synthesis and cell division in *D. vulgaris* (Clark et al., 2012; Egan et al., 2017). It was concluded that *D. vulgaris* cells use phospholipids released from neighboring dead cells for synthesizing amino acids. The proteins that impact carbohydrate and nitrogen metabolism (Barton and Hamilton, 2007) have been observed and proposed by several researchers. Prior studies have depicted lower carbohydrate content in SRB biofilm than planktonic cells (Clark et al., 2012; Qi et al., 2016). In the study by Qi et al. (2016), the protein content in DVH biofilm cells was almost similar to planktonic cells after 5 days of growth. However, the carbohydrate content in the biofilms decreased significantly at the end of the fifth day, which resulted in a low carbohydrate to protein ratio of 0.11. These results corroborate that SRB biofilms do not produce a carbohydrate-rich biofilm matrix (Clark et al., 2007). In addition to dehydrogenases, fructose-1,6-bisphosphatase was also found downexpressed in DVH biofilm, indicating a decrease in gluconeogenesis rate (Clark et al., 2012) and supporting the hypothesis of slow cell wall synthesis and cell division.

The nitrogen metabolism in SRB allows adaptation in different nutritional habits even under adverse conditions (Lespinat et al., 1987). There is limited information available regarding nitrogen metabolism in SRB biofilm. The studies that focused on transcriptional heterogeneity have found increased expression of *nrfA* gene (cytochrome c nitrite reductase c552) in the presence of ammonium in the biofilm, but upregulation was also reported when cells were exposed to acetone, ethanol, nitrite, and alkaline pH. A *nifU* (nitrogenase) gene was found in DVH plasmid, but no change in expression has been recorded (Clark et al., 2012). Therefore, it can be concluded that nitrogen metabolism is still not well understood in SRB biofilms, and the expression of *nrfA* may be a response to general stress management. More studies are required to unveil the nitrogen metabolism in SRB biofilm.

#### Genes and Proteins Involved in Stress Response

Biofilm formation involves stress response regulators, and different stress conditions trigger changes in the expression of genes that help microbes survive. The differential expression of the stress response regulators depends upon the environments under which biofilm formation is triggered and the genetic background of the microbe (Jefferson, 2004). Here, we review how different environmental parameters (media type, nutrient deprivation, pH, oxygen radicals, osmotic stress, temperature, and material surface) influence the formation of SRB biofilm. It is possible to infer that the relative expression of various stress-related genes and proteins varies accordingly. For instance, in DVH, the stress response gene DVU2410 coding for superoxide dismutase had increased transcript level (Clark et al., 2012), whereas the same gene was downexpressed in a study reported by Qi et al. (2016). These two studies primarily differed in the type of media and incubation temperature. Clark et al. (2012) used lactate C media (*T* = 30°C), while the latter used LS4D media and different incubation temperatures (*T* = 35°C). In addition, a comparison of differential gene expression in other studies also corroborates this hypothesis. For example, mRNA levels for various stress response genes were elevated in the biofilms of certain microorganisms (e.g., *Escherichia coli*, *Pseudomonas aeruginosa*, and *Staphylococcus aureus*). In contrast, for the anaerobic, hyperthermophile *Thermotoga maritima*, a decrease in the expression of similar genes was documented (Schembri et al., 2003; Pysz et al., 2004; Waite et al., 2006). It is also worth mentioning that the work of Qi et al. (2016) was done on single cells while the transcriptomics analysis of Clark et al. (2012) was the average across biofilm cells in the sample.

For DVH grown in LS4D media at 30°C, various chaperone genes such as *uspA* (DVU0423) and three *hsp20* genes (DVU2442, 2241, and 1471) had been reported with increased expression within the biofilm compared to planktonic cells (Clark et al., 2012). An increase in expression of alkyl hydroperoxide reductase *ahpC* (DVU2247), *msrB* (DVU0576), and *ahpC/TSA* (thiol-specific antioxidant) was also seen in the same study. In addition, relatively slower growth is known for aerobic bacteria due to oxidative stress within the biofilm due to a shift away from oxygen respiration at different biofilm depths (Stewart and Franklin, 2008). However, slower biofilm growth kinetics is attributed to lower intracellular levels of reducing equivalents for SRB biofilms. This condition might make SRB more susceptible to oxidizing agents, and therefore, a defense mechanism is triggered, resulting in overexpression of genes and proteins involved in potential detoxification. For instance, the rubrerythrin gene (*rbr*) and thioredoxin reductase (*trxB*) were upregulated in response to oxidative stress, suggesting an important role of these proteins in the oxidative damage resistance response in DVH (Zhang et al., 2006). The upregulation of *trxB* suggests that thiol-specific redox system might be involved in the oxidative stress response in SRB. Pereira et al. (2008) reported an upregulation of proteins (the [NiFeSe] hydrogenase, formate dehydrogenase(s), and the Hmc membrane complex) associated with periplasmic hydrogenase-dependent mechanism during oxidative stress in DVH. An increase in expression levels for genes and proteins involved in potential detoxification was also noticed during iron limitation due to sequestration and intracellular accumulation of iron (Hindré et al., 2008; Clark et al., 2012). This increase in the expression of detoxification genes and proteins may be due to the ability of some metals to form hydroxyl radical, metallo-oxo, and metallo-peroxo species (Kasprzak, 2002). In addition, genes annotated as proteases such as ion protease (DVU1337), *clpX* (DVU1336), *clpB* (DVU1874), *htrA* peptidase (DVU3278), *htpG* (DVU2643), and *hflC* (DVU0683) were downregulated (Clark et al., 2012). One potential reason for this might be a slower turnover of proteins due to the biofilm growth, but more research is needed to confirm this behavior. DVH biofilm cells also showed altered transcript expression profiles for methyl-accepting chemotaxis proteins, histidine kinases, response regulators, and sensory box proteins, as shown in Table 1.

#### Genes and Proteins Involved in Iron Acquisition and Transporters

Iron uptake by SRB is necessary for maintaining an optimal concentration of cellular biomass and enzymatic activity. A previous study found that removing iron from the culture medium resulted in a decrease in the concentration of cellular biomass, and adding back iron to the culture medium restored a high level of biomass concentration (Marchal et al., 2001). DVH biofilms are predicted to be iron limited, as shown by the increase in expression of iron transporter subunit *feoA* (DVU2572) and *fepC* (DVU0103), a presumptive ABC transporter for iron uptake (Clark et al., 2012). However, similar findings in planktonic cells (Clark et al., 2006) contradict these results. One plausible reason might be that iron acquisition response is a consequence of increased sulfide concentration associated with SRB growth (Clark et al., 2012). In addition, Clark et al. (2012) found that iron storage protein bacterioferritin (DVU1397) and ferritin storage protein (DVU1568) were upregulated within DVH biofilm cells. The upregulation of iron sequestration proteins was also reported in similar biofilm studies (Pysz et al., 2004; Zhang et al., 2007; Hindré et al., 2008). However, under conditions when DVH biofilm was grown on steel, the iron transport systems like *feoAB* were downregulated (Zhang et al., 2007). Available iron acts differently in the biofilm of different organisms. In some, it induces a thick biofilm formation (Banin et al., 2005), while in others, it plays a detrimental role (Johnson et al., 2005; Hindré et al., 2008).

Among other transporters, Clark et al. (2012) found that four presumptive ABC transporter genes (DVU2387, DVU2384, DVU0484, and DVU2385) were upregulated in DVH biofilm. Other studies on biofilms from different organisms have also reported upregulation of ABC transporter genes (Schembri et al., 2003; Pysz et al., 2004; Shemesh et al., 2007). Subunits of the Sec transport system [*secE* (DVU2922), *secY* (DVU1323), and *secG* (DVU1676), and *secD* (DVU1819)] responsible for protein secretion across the cytoplasmic membrane were transcriptionally downexpressed at the protein level (Clark et al., 2012; Pena et al., 2019). However, other types of secretion systems have been reported to be amplified, as indicated by the increased abundance of transcripts of a type I system (DVU1013) and increased protein levels of an annotated type III protein secretion system (DVUA0115) (Clark et al., 2012). The role of DVUA0115 has been proven when corresponding gene (present on plasmid pDV1 of DVH) deficient strain (plasmid cured) lost the ability to form biofilm (Clark et al., 2007).

#### Genes and Proteins Involved in Extracellular Proteins, Fatty Acid, and Lipid Synthesis

*Desulfovibrio* biofilms are a complex system with hundreds of genes that are up-/downregulated during the biofilm advancement process. The extracellular proteins present in the biofilm matrix contribute to biofilm structure and stability. Mutational studies have shown that the absence of extracellular proteins in the biofilm matrix results in reduced biofilm formation, stability, and altered biofilm architectures (Kobayashi and Iwano, 2012). The von Willebrand factor domain (vWF) glycoprotein performs various roles in conjunction with different ligands that include cell adhesion, pattern formation, and signal transduction (Colombatti et al., 1993). Clark et al. (2012) found DVU1012 (have a vWF domain) as most abundant and also contain a hemolysin-type calcium-binding domain. The paralog of DVU1012 and DVU1545 was also abundant in extracellular proteins from the biofilm. They also found that two hypothetical/presumptive proteins (DVU0797 and DVU0799) showed increased abundance in both the extracellular and biofilm fraction of proteins but not in mRNA. A study by Zeng et al. (2017) found that both DVU0797 and DVU0799 code for porin proteins. This highlights that the participation of porin proteins in extracellular matrix formation in biofilm structures could play a potential role.

Lipids, including triglycerides and fatty acids, are the main constituents of the cytoplasmic membrane. They are indispensable for bacterial integrity, survival, and growth. Thus, they may play an important role during biofilm formation (Dubois-Brissonnet et al., 2016). However, there are contradicting reports on the fatty acid composition of DVH biofilm. In one study, *lpxC* (lipid A production), *TolB* (peptidoglycan-associated lipoprotein), and DVU0869 (undecaprenyl diphosphate synthase) were downregulated (Clark et al., 2012). The downregulation of fatty acid synthesis genes was contradicted in a separate study, where fatty acid biosynthesis in the biofilm cells was upregulated in DVH biofilm cells (Zhang et al., 2016). These contrasting results may be due to the differences in the environment conditions (temperature, pH, and media composition) and the time at which samples were collected. As such, more insights into the fatty acid regulation of DVH biofilm cells are required.

### Genes and Proteins Regulating Microbiologically Influenced Corrosion and Biofilm Formation

Microbiologically influenced corrosion results from biochemical reactions that release ions or electrons from the metal surface. Microbes stimulate corrosion through the secretion of enzymes, acidic metabolites, and hydrogen consumption that stimulates cathodic reactions (Kip and van Veen, 2015). SRB are anaerobic microorganisms that have been implicated in the corrosion of iron and steel. In the study by Caffrey et al. (2008), the gene expression profile of DVH was analyzed by growing it on iron under cathodic protection conditions (cathodic hydrogen) and comparing it with cells grown with gaseous hydrogen bubbling through the culture. The cells grown under cathodic protection conditions overexpressed two hydrogenases, the *hyn-1* genes for [NiFe] hydrogenase and the *hyd* genes encoding [Fe] hydrogenase. The electron flow from the hydrogenases across the cytoplasmic membrane was also enhanced because the *hmc* gene encoding high molecular weight cytochromes was overexpressed under the cathodic protection conditions. The corrosion rate of iron electrodes was significantly reduced when *hyn-1*, *hyd*, and *hmc* mutant biofilms were grown, suggesting that these three genes contribute to the corrosion-enhancing metabolism in DVH. Motility is another critical factor influencing biofilm architecture, specifically for overcoming stressful conditions (O’Toole et al., 2000; Houry et al., 2012). Flagellar proteins have been reported to play an essential role in triggering the initial syntrophic interactions (with other microbes) and fitness for biofilm formation (Shimoyama et al., 2009; Williamson et al., 2018). Krumholz et al. (2015) found that DA-G20 mutated for two flagellum genes (*flhA* and *fliF*) exhibited no motility, and also the mutant was unable to utilize the source of H_2_ in media. Furthermore, significantly decreased biofilm formation was observed in *tadC* (pilus assembly protein) and glycosyltransferase mutants of DA-G20. Mutant of the gene Dde_0681 (Fe–S cluster hydrogenase component) was incapable of syntrophic coupling due to lower growth on H_2_ or lactate with sulfate as electron acceptor; however, the growth was normal with thiosulfate or sulfite as electron acceptors. Mutant for Fe-only hydrogenase, the quinone reactive complex (*qrc*), and high molecular weight cytochrome C complex (*hmc*) was found to be syntrophically deficient and had almost no growth with H_2_/sulfate. Out of the three periplasmic hydrogenases, only the two Ni–Fe hydrogenases (Dde_2137-38 and Dde_3754-56) were found to be significantly upregulated.

In the study by Wikieł et al. (2014), metabolic activity and biofilm development of DA-G20 was correlated to electrochemical response on carbon steel surfaces. Response regulators/GGDEF domain protein in DA-G20 has an essential role in biofilm formation. A cyclic-di-GMP signaling network regulates various bacterial groups’ biofilm formation and motility (Römling et al., 2013). DVH genome encodes c-di-GMP-modulating response regulators, which are part of a two-component signaling system and confirmed that a transposon mutant (DVU0636: a c-di-GMP-modulating response regulator) strain of DVH lost its ability to form biofilm. The biofilm demonstrated an altered carbohydrate:protein ratio compared to the wild-type strain (Rajeev et al., 2014). In addition, type IV pili also plays a vital role in adherence of cells as an initial step in colonization (Craig et al., 2019), and *pilA* mutant (lacking a presumptive type IV pilus) in DVH lost its ability to form a biofilm (Brileya et al., 2014). However, the wild-type DVH strain used by Brileya et al. (2014) and Rajeev et al. (2014) is designated as DVH-MO (strain housed at University of Missouri), and the discovery of biofilm deficiency in DVH-MO might change the interpretation of the involvement of these genes (*pilA*, *flhA*, *fliF*, and *tadC*) in biofilm formation (De León et al., 2017). The DVH-MO strain has a single nucleotide change within the gene DVU1017 [ABC transporter of a type I secretion system (T1SS)], which was discovered to be sufficient in eliminating biofilm formation.

**FIGURE 1:**
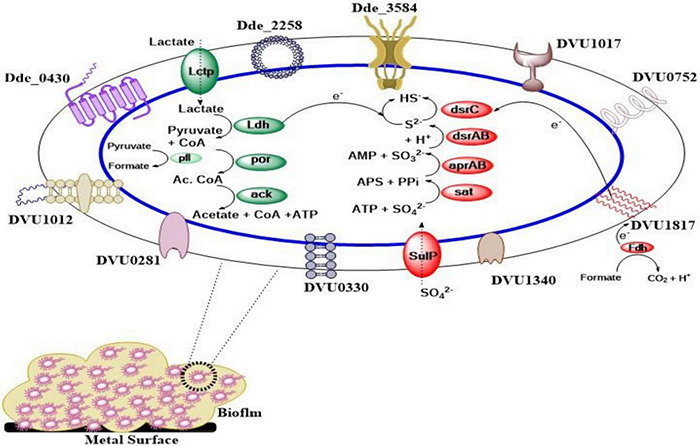
Pictorial representation of biofilm forming and lactate oxidation/sulfate reduction proteins. (Cellular location of all the biofilm formation genes is shown in Supplementary Table 1. *Lctp*, lactate permease; *Ldh*, lactate dehydrogenase; *por*, pyruvate-ferredoxin oxidoreductase; *ack*, acetate kinase; *pfl*, pyruvate formate lyase; *SulP*, sulfate permease; *sat*, sulfate adenylyltransferase; *aprAB*, adenosine-5′-phosphosulfate (APS) reductase; *dsrAB*, dissimilatory sulfate reductase; *dsrC*, dissimilatory sulfite reductase (*Desulfoviridin*), gamma subunit; *Fdh*, formate dehydrogenase).

In addition to c-di-GMP-based regulation, sigma factors (σ factors) regulate bacterial transcription and control how efficiently transcription is initiated (Francke et al., 2011; Bush and Dixon, 2012). There are different classes of σ factors present in bacteria, as described previously (Burgess, 2017). In general, σ factors are subunits of all bacterial RNA polymerases (RNAPs) that determine transcriptional initiation specificity through binding to RNAP (Kazakov et al., 2015). One of the sigma factors, called alternative sigma factor σ54, constitutes 41% (37 out of 91) of all response regulators (RR) in DVH when compared to other bacteria where only 10% RR are σ54 dependent (Kazakov et al., 2015; Zhu et al., 2019). The 37 regulons that σ54-dependent regulators control in DVH are involved in nitrogen, carbon, and energy metabolism; transmembrane transport; nitrite stress response; and exopolysaccharide and biofilm synthesis (Kazakov et al., 2015). Zhu et al. (2019) found that in DVH, σ54-dependent regulator (DVU2956) is highly upregulated in planktonic cells and downregulated in biofilm. The σ54-dependent regulator (DVU2956), when expressed in DVH biofilm through the use of promoter that is exclusively expressed in the biofilm mode of growth, reduced biofilm formation by 72 ± 11% in 48 h. On the contrary, knocking out DVU2956 increased biofilm formation by 30.1 ± 0.6%. In addition to suppressing biofilm formation, Zhu et al. (2019) also found that DVU2956, when produced 24 h after biofilm formation, was able to disperse DVH biofilm by 42 ± 4%. Apart from DVH, when the plasmid-expressing DVU2956 was introduced in DA-G20, the biofilm was inhibited by 78 ± 9% after 24 h. This shows that DVU2956 can inhibit biofilm formation in multiple SRB strains. Figure 1 represents a pictorial representation of lactate oxidation and sulfate reduction along with proteins involved in biofilm formation and their cellular location in DA G20 and DVH.

## Comparative Genomics of Sulfate-Reducing Bacteria

**FIGURE 2:**
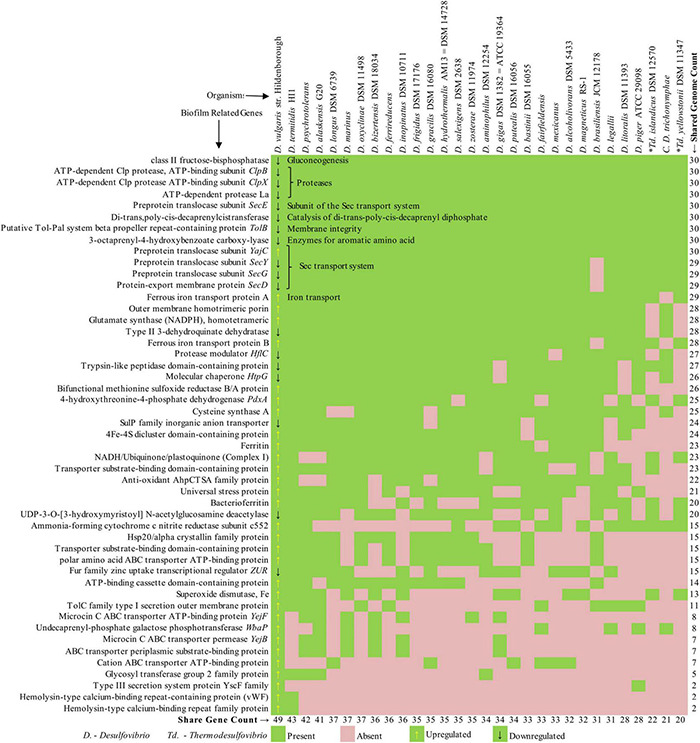
Presence–absence matrix of biofilm genes shared across 30 sulfate-reducing bacteria (SRB) genomes.

The previous section discussed the genes in different functional categories that take part in critical cellular processes and stress response systems during biofilm formation by SRB. These genes were either up- or downregulated during the biofilm formation, indicating their role in biofilm development. This section has compiled a list of the reported biofilm-related genes from prior studies and mapped it across 30 common SRB based on sequence similarity (Supplementary Table 1). The organisms were selected based on the availability of quality genomes from the National Center for Biotechnology Information (NCBI). The heatmap was generated based on the presence/absence matrix of the genes, as shown in Figure 2. The genes are organized from top to bottom in the order of presence across the genomes, whereas the genomes are arranged from left to right in the order of the number of shared genes. This means that the organisms on the left-most side of Figure 2 have the maximum number of gene features, and the gene listed on the top is shared by most organisms. The first column of the plot is marked with an up- and downregulated sign. As depicted in the plot, only nine genes are shared across all the SRB, indicating its role in essential functions. The respective functions (gluconeogenesis, protein catalysis, sec transport system, and membrane integrity) are mentioned inside the heatmap.

Interestingly, eight of the nine genes (except *YajC*) are reported to be downregulated during the biofilm formation (Clark et al., 2012). As we move down, the list of genes that are shared by a smaller number of organisms is mainly upregulated. Expectedly, the closely related organisms (DVH, DA-G20) shared most of the genes and are positioned on the left half of the plot. On the contrary, the distant organism (*Thermodesulfovibrio*, *Candidatus Desulfovibrio*) shared the minimum number of genes and are positioned on the right half. Despite the organisms share many of the listed genes, whether they are up- or down-regulated depends on several unknown factors. This comparative study demonstrates the distribution of active biofilm genes across the genomes of a selected set of sulfate-reducing bacteria, which might be helpful to predict the biofilm nature of other SRB.

## Role of Quorum Sensing in Sulfate-Reducing Bacteria Biofilm Formation

The presence of biofilm facilitates direct contact between the metal surface and SRB outer membrane redox proteins or with electroconductive filaments (Beese-Vasbender et al., 2015). Such biofilm matrix establishes anoxic niches in which SRB proliferate, resulting in localized corrosion and pits. The biofilm matrix primarily comprises of rhamnolipids and extracellular polymeric substances (EPS), and the genes responsible for these are controlled by the quorum-sensing (QS) system (Scarascia et al., 2019). QS is a cell-density-dependent communication system in microbes based on the exchange of small molecules called autoinducers. When the density of cells reaches a threshold, such autoinducers bind to receptors to initiate a cascade of reactions that leads to an increase in biofilm formation by overexpressing or suppressing the expression of certain genes (Waters and Bassler, 2005). The molecules used for QS are highly specific, produced and recognized by many different bacterial species. In general, Gram-negative bacteria use small QS molecules known as autoinducers {autoinducer-1 [acyl-homoserine lactone (AHL)] and autoinducer-2}, while Gram-positive bacteria use oligopeptides as QS molecules (Mukherjee and Bassler, 2019). However, studies have detected autoinducer-2 type QS molecules in Gram-positive bacterial species (Surette et al., 1999; Pereira et al., 2013). The presence of autoinducer 2 QS signal molecule in both Gram-positive and Gram-negative bacteria helps mediate interspecies communication between diverse bacterial species (Zhang et al., 2020).

Although the complete mechanism of QS in SRB is still unknown, inferences on the presence of putative QS systems can be made through *in vitro* cell assay and comparative genomic analysis (Kawaguchi et al., 2008; Scarascia et al., 2016). Some studies using an *in vitro* cell-free assay have demonstrated the presence of several AHL molecules in active cultures of *D. vulgaris* and other *Desulfovibrio* spp. (Kawaguchi et al., 2008; Decho et al., 2010; Scarascia et al., 2016). Diverse AHLs were also detected in a study on the examination of QS molecules in microbial mats with a high abundance of SRB (Decho et al., 2009). Previous studies have suggested that long-chain alkyl AHLs produced by SRB stimulated sulfide oxidation by sulfide-oxidizing bacteria (SOB) (Montgomery et al., 2013). This syntrophic behavior helps SRB generate ATP by gaining access to sulfate through SOB(Scarascia et al., 2016). Genomic approaches have found proteins homologous to *LuxR* and *LuxS* in *Desulfovibrio magneticus*, *Desulfovibrio desulfuricans*, and *DVH* (Brady et al., 2004; Williamson et al., 2005; Kimura, 2014). In addition, genome mining of SRB genomes showed that QS proteins homologous to *LuxS*, *LuxP*, *LuxQ*, and *LuxO* are present in many *Desulfovibrio* spp. Interestingly, *LuxO* is reported to be involved in the downstream phosphorylation cascade reactions to upregulate or repress QS-associated genes involved in many phenotypic traits, including biofilm formation (Scarascia et al., 2016). Data mining results from a previous study (Table 2) confirm the presence of QS systems in SRB similar to that present in *Vibrio harveyi* (Scarascia et al., 2016). In a recent study, AHLs and autoinducer-2 QS molecules were detected in the culture of *Desulfovibrio* sp. Huiquan 2017 (Li et al., 2021). The presence of autoinducer-2 in other non-desulfovibrio Gram-negative SRB (e.g., *Desulfobacter postgatei*, *Desulfobacterium autotrophicum*, and *Desulfobulbus propionicus*) signifies the use of autoinducer-2 as signal molecule mediating interspecies communication among bacteria. In addition, Gram-positive *Desulfotomaculum* genomes showed that the majority of them (e.g., *Desulfotomaculum kuznetsovii*, *Desulfotomaculum ruminis*, *Desulfotomaculum reducens*, and *Desulfotomaculum acetoxidans*) have *LuxR*-type QS proteins (Scarascia et al., 2016). There are two plausible explanations for the presence of *LuxR*-type QS proteins in Gram-positive SRB: (1) these proteins may merely be orphan receptors, which have no signal to respond to and therefore are not involved in QS, and (2) these orphan receptors might allow other SRB to sense and respond to QS signal produced by other bacteria present within the microbial community. However, further research is required to prove either of these hypothesis and to establish a direct link between QS in SRB and sulfate reduction and subsequently MIC.

**TABLE 2:** Quorum sensing (QS) protein homologs in SRB as compared to *Vibrio harveyi.*

**SRB**	**QS protein in *Vibrio harveyi***	**Homologous protein in SRB database**	**Query cover (%)**	***E*-value**
*Desulfovibrio salexigens*	LuxS	Quorum sensing AI-2, LuxS	100	2E-18
*Desulfovibrio alaskensis*	LuxP	AI-2 binding perisplatic protein, LuxP precursor	89	2E-105
*Desulfotomaculum nigrificans*	LuxR	2 components transcriptional regulator, LuxR family	99	5E-42
*DVH*	LuxO	Sigma54 specific transcriptional regulator	53	4E-82
*Desulfovibrio hydrothermalis*	LuxS	*S*-ribosylhomocysteine lyase	100	3E-19
*DVH*	LuxR	LuxR family transcriptional regulator	93	2E-51
*Desulfovibrio hydrothermalis*	LuxQ	Signal transduction histidine kinase	31	5E-23
*Desulfovibrio salexigens*	CqsS	PAS/signal transduction histidine kinase	52	1E-83
*Thermodesulfovibrio aggregans*	LuxR	LuxR family transcriptional regulator	99	3E-36
*Desulfotignum phosphitoxidans*	LuxO	Luminescence regulatory protein, LuxO	67	7E-85
*Desulfovibrio piezophilus*	LuxP	AI-2 binding perisplatic protein, LuxP	95	7E-95

*DVH, *Desulfovibrio vulgaris* Hildenborough.*

## Mechanism of Microbiologically Induced Corrosion

### Cathodic Depolarization and Biocatalytic Sulfate Reduction

**FIGURE 3:**
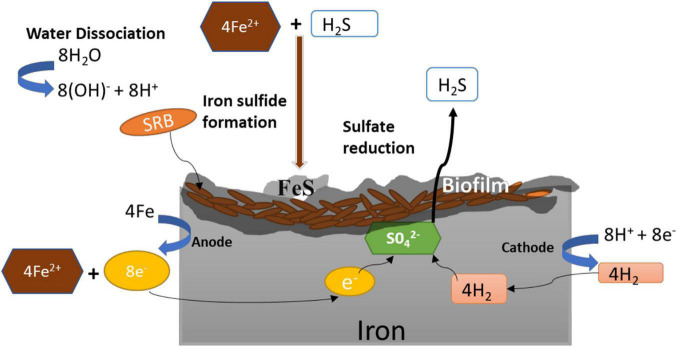
Mechanism of microbiologically influenced corrosion (MIC) of iron by sulfate-reducing bacteria.

Microbiologically influenced corrosion (a.k.a. biocorrosion) results from a series of coordinated interactions among the bacterial cells and the underlying metal interfaces (Beech and Sunner, 2004) that may generate corrosive metabolites that accelerate corrosion. Due to the generation of corrosive metabolite (hydrogen sulfide), MIC is also known as metabolite corrosion or chemical microbially influenced corrosion (CMIC), whereas corrosion through direct withdrawal of electrons is referred to as electrical microbial influenced corrosion (Enning and Garrelfs, 2014). SRB are non-pathogenic bacteria that use self-secreted enzymes for accelerating the sulfate reduction that indirectly promote oxidation of the underlying metals. Mild steel, stainless steel, carbon steel, copper, and aluminum are examples of industrially relevant metals vulnerable to MIC (Kakooei et al., 2012). Kuehr and Vlugt (1934) first explained the MIC mechanisms using cast iron as model substrates. The proposed mechanism was based on cathodic depolarization (CDP) caused by the hydrogenase-catalyzed reduction of sulfate (SO${}_{4}^{2-}$) by H_ads_ (Figure 3). The increased consumption of cathodic hydrogen increased cathodic reactions leading to high corrosion rates. In CDP theory, water was first partially dissociated into hydrogen and hydroxyl ions (Equation 1), and the exposed cast-iron surfaces were ionized by the electrolytic solution pressure to form ferrous ions (Equation 2). The protons released by dissociation of water (Equation 1) were discharged on the cathode, where they react with electrons (Equation 2) to form hydrogen (H_ads_) that polarizes the cathode. The hydrogenase enzyme converts H_2_ to H^+^ (Equation 4), and the sulfate that acts as depolarizer was reduced by the free electrons and protons (Equations 1 and 2) to generate hydrogen sulfide (Equation 5), which provides energy for SRB metabolism. In the subsequent precipitation reactions (Equations 6 and 7), ferrous ions react with hydrogen sulfide and hydroxyl ions to form FeS and Fe(OH)_2_.

**Table:** 

Water dissociation:	8H_2_O → 8H^+^ + 8OH^–^	(1)
Anodic reaction:	4Fe + 2H → 4Fe^2+^ + 8e^–^	2)
Cathodic reaction:	8H^+^ + 8 e^–^ → 8H_ads_	(3)
Cathodic depolarization by hydrogenase:	8H_ads_ → (4H_2_) → 8H^+^ + 8 e^–^	(4)
Sulfate reduction by SRB	SO_4_^2–^ + 8e^–^ + 9H^+^ → HS^–^ + 4H_2_O	(5)
Precipitation:	Fe^2+^ + H_2_S → FeS + 2H^+^	(6)
Precipitation:	3Fe^2+^ + 6OH^–^ → 3Fe(OH)_2_	(7)
**Total reaction:**	**4Fe + SO_4_^2^**^–^ **+ 4H_2_O** → **FeS + 3Fe(OH)_2_ + 2OH**^–^	(8)
		

Although the CDP theory (Kuehr and Vlugt, 1934) has been questioned, it is still used to describe SRB-induced corrosion mechanism (Procópio, 2019). The primary basis for questioning the CDP mechanism is that the rate-determining step for hydrogen evolution on iron is adsorption rather than desorption (Blackwood, 2020). This means that removing H_2_ from the surface is not likely to accelerate the cathodic reaction, as it is not the rate-limiting step (Frankenthal and Milner, 1986). In addition, many studies have concluded that both hydrogenase^+^ and hydrogenase^–^ SRB affect corrosion rate in similar ways (Hardy, 1983; Dinh et al., 2004; Mori et al., 2010). Despite issues with the original CDP theory, cathodic depolarization is consistently observed during corrosion by SRB, alluding to the fact that the kinetics of cathodic reaction rates was indeed increased. This was explained by King and Miller (1971), who suggested that the formation of a conductive film of iron sulfide, which is catalytic to the reduction in protons, results in a positive shift in corrosion potential leading to cathodic depolarization. Furthermore, as most metal sulfide films are cathodic to the base metal, any damage to the film exposes the metal creating localized cathodic and anodic sites (leading to galvanic corrosion) (Syrett, 2013). In addition, microbes in a biofilm arrange themselves in a mutualistic manner that imposes chemical gradients, which creates microenvironments of oxygenated and deoxygenated niches on the metal sulfide layer. The cycling between oxygenated and deoxygenated environments increases corrosion rates, since metal sulfides may act as catalysts to reduce oxygen (Hamilton, 2003; Sherar et al., 2013). The reduction in oxygen by metal sulfides inhibits the formation of the normal protective layer of metal oxides. The erosion of metal oxides due to the reaction between oxygen and metal sulfides exposes anodic sites and initiates pitting corrosion (Hardy and Bown, 1984; Hamilton, 2003).

Another mechanism of biocorrosion reported in 2009 was based on the biocatalytic cathodic sulfate reduction theory (BCSR) (Gu et al., 2009). The BCSR theory analyzed MIC by SRB on carbon steel by combining the concepts of bioenergetics and electrochemical kinetics. Here, MIC is induced by accelerated sulfate reduction at the cathode by the electrons released through iron dissolution at the anode. Under anaerobic conditions, SRB forms biofilm on the surface of the iron. In the initial growth phase under standard conditions, SRB couples oxidation of C substrate such as lactate [e^–^ donor, *E*°′ = −430 mV vs. SHE (standard hydrogen electrode)] with the reduction in sulfate (e^–^ acceptor, *E*°′ = −213 mV vs. SHE) (Gu et al., 2009). The standard Gibbs free energy change of the two reactions (ΔG°′ = −168 kJ/mol sulfate) implies that the redox reaction is thermodynamically feasible. SRB couple these two reactions to derive metabolic energy for their growth and metabolism. When the SRB cells evolve to grow into a dense biofilm, the monolayer of cells at the bottom of the biofilm is deprived of C source (e^–^ donor), which results in C starvation. In order to meet their maintenance energy, SRB extracts electrons from elemental iron instead of organic carbon. Thus, the C oxidation is replaced by the elemental iron oxidation with the following reactions (Kakooei et al., 2012).

The increase in corrosion rate due to C starvation is only applicable to metals with iron as the base material (carbon steel and stainless steel). Unlike carbon and stainless steel, C starvation reduced copper corrosion rates by DVH (Dou et al., 2018). The experimental data analysis and thermodynamic study indicated that MIC of Cu by *D. vulgaris* during C starvation was caused by secreted sulfide as compared to electron harvesting from metal surface in the case of iron. Another study on copper alloy (70Cu–30Ni) found that polarization resistance was reduced for copper alloy in the presence of SRB (Huang et al., 2004). The H_2_S produced by SRB lowered the reduction potential of anodic reaction, which resulted in an increase in corrosion rate. Moreover, the formation of Cu_2_S film accelerated localized corrosion due to increased heterogeneity of the copper surface (Dou et al., 2018). In addition to copper and its alloys, aluminum alloys are also widely used in industries and marine engineering facilities (Varshney and Kumar, 2021). Guan et al. (2020) studied the effect of two aluminum alloys (5,052 and Al–Zn–In–Cd aluminum alloy) on SRB metabolic activities and found that both alloys promoted SRB metabolic activity. Compared with 5,052 aluminum, which is known to have good anticorrosion characteristics due to the formation of passive film on its surface, the Al–Zn–In–Cd aluminum alloy is quite active in salt water. In the presence of SRB, the [H] generated at the cathode acts as an electron transfer mediator in the process of EET used by the SRB. This increases cathodic reaction rate due to a large number of H leaving the metal surface. At the same time, the anodic reaction remains stable, which causes electron dissipation from the metal surface (Yu et al., 2013; Guan et al., 2020). The EET mechanism of SRB transports extracellular electrons to the cells cytoplasm for reduction reaction, which indicates that SRB cell acts as biocathode and the bacterial cells directly obtain electrons. This EET mechanism increases corrosion potential of Al–Zn–In–Cd aluminum alloy, which accelerates corrosion rates. The role of EET in corrosion is discussed in detail in the next section.

**Table:** 

Anodic:	4Fe^2+^ + 8e^–^ → 4Fe (Iron Dissolution)	*E*°′ = −447 mV
Cathodic:	SO_4_^2–^ + 9H^+^ + 8e^–^ → HS^–^ + 4H_2_O (BCSR)	*E*°′ = −213 mV
**Total:**	**SO_4_^2^**^–^ **+ 9H**^+^ **+ 4Fe** → **4H_2_O + HS**^–^ **+ 4Fe^2^**^+^	*E*_cell_ = +234 mV

### Role of Extracellular Electron Transfer in Microbiologically Influenced Corrosion

Extracellular electron transfer (EET) is a mode of energy conservation by which some microorganisms exchange intracellular electrons with extracellular electron donor/acceptor (Rathinam et al., 2018; Tanaka et al., 2018; Shrestha et al., 2020). Microbial EET potentially endangers corrosion of iron structures. Microbial EET activity augments corrosion *via* (1) direct uptake of electrons from metallic iron, also known as direct electron transfer MIC (DET-MIC) (Xu et al., 2013; Kato, 2016), and (2) indirect transfer of electrons through the use of electron carriers (mediators), known as mediated electron transfer (MET) (Xu et al., 2013; Zhang et al., 2015). EET microorganisms capable of inducing corrosion are isolated from marine environments and are primarily identified as SRB and methanogenic archaea (Kato et al., 2015). SRB are one of the most prominent microorganisms known to cause anaerobic MIC, mainly *via* the production of corrosive chemical H_2_S. However, a number of SRB and other bacterial strains stimulate corrosion *via* more direct manners. For instance, Jia et al. (2018) reported that lower H_2_S concentration in the culture medium inoculated with *D. vulgaris* (ATCC 7757) increased MIC rate on C1018 carbon steel. It was possibly due to the reduction in acidity as the concentration of biogenic H_2_S in the culture medium decreased. On the contrary, the presence of more biogenic H_2_S in the culture medium increased acidity, decreased sessile cell count on iron surface, and formed a protective film of Mackinawite on iron surface, thereby inhibiting corrosion. Furthermore, it was also reported that, at pH above 7, direct electron uptake by sessile SRB cells is the dominant mechanism of corrosion.

Some anaerobic microorganisms use DET to utilize zero-valent metallic iron as their electron donor, thereby stimulating iron corrosion. Anaerobic iron corrosion has frequently been reported, and in most cases, it is linked to SRB activity (Dinh et al., 2004). Accelerated cathodic reaction *via* the consumption of cathodic electrons (cathodic depolarization) is one of the significant causes of MIC. Cathodic depolarization is induced by microbes consuming abiotically generated H_2_ or by methanogenesis. However, the stimulation of anaerobic corrosion *via* H_2_ consumption is controversial. It has been reported that H_2_ consumption by itself is not sufficient to induce fatal iron corrosion (Blackwood, 2020). A recent study reported that there is no abiotic generation of H_2_ from stainless steel, further demonstrating that the corrosion mechanism involving H_2_ as electron carrier is dubious (Tang et al., 2021). Studies have shown that only microorganisms with EET ability (direct or indirect uptake of electrons from metallic iron) can stimulate cathodic reaction and corrode iron aggressively (Venzlaff et al., 2013; Blackwood, 2020; Tang et al., 2021). This manner of MIC, i.e., stimulation of the cathodic reaction by consuming cathodic electrons as the metabolic energy source, is known as electrical MIC (Enning and Garrelfs, 2014). Tang et al. (2021) reported that two *Geobacter* species (*Geobacter sulfurreducens* and *Geobacter metallireducens*) were able to directly extract electrons from stainless steel *via* DET between the bacteria and iron. When the gene (*Gmet 1668*) coding for multi-heme c-type cytochrome, which is required for DET, was knocked out, the pitting corrosion drastically reduced. *Gmet 1668* could be used in future studies as a molecular target to find microbes with DET capability. Similarly, other SRB strains such as *Desulfopila corrodens* strain IS4 and *Desulfovibrio ferrophilus* strain IS5 reduce sulfate with concomitant oxidation of metallic iron much faster than abiotic H_2_ generation in an organic matter-free medium (Kato, 2016). Compared to DET, Zhang et al. (2015) observed that MET in the presence of electron mediators (10 ppm) such as riboflavin and flavin adenine dinucleotide (FAD) enhanced pitting corrosion of 304 stainless steel by *D. vulgaris* (ATCC 7757). C-source starvation also plays an important role in electron extraction from iron surface (Xu and Gu, 2014). During C-source starvation, the sessile cells at the bottom might not receive adequate carbon for their survival. As a consequence, the cells at the bottom switch to Fe^0^ as the terminal electron donor, and the electrons thus released are transported by EET (DET or MET) into the cytoplasm where sulfate reduction takes place. It has been shown that *D. vulgaris* (ATCC 7757) biofilm grown on iron surface with 90% carbon reduction increased corrosion rate causing a 10-μm maximum pit depth (Xu and Gu, 2014). In addition, the effect of sulfate concentration on biofilm aggressiveness was depicted in a mechanistic modeling study of MIC by SRB (Xu et al., 2016). Sulfate reduction depends on the ability of an SRB biofilm to transfer extracellular electrons from iron surface to SRB cytoplasm. The ability of an SRB biofilm to transfer extracellular electrons is dependent on the type of EET mechanism employed (DET or MET). However, mass and charge transfer becomes increasingly limiting because sulfate has to diffuse through a much larger distance to the bottom of biofilm to get reduced by extracellular electrons. The mechanistic modeling results by Xu et al. (2016) indicated that at high sulfate concentration (7–28 mM), charge transfer becomes limiting, and pitting corrosion depth diminishes. On the other hand, at low sulfate concentration (∼1 mM), mass transfer becomes limiting, and pit growth starts to grow more prominently. So far, EMIC-inducing abilities in SRB have been identified in only a limited number of strains in the deltaproteobacterial families *Desulfovibrionaceae* and *Desulfobulbaceae* (Enning and Garrelfs, 2014). Many SRB cannot grow autotrophically and require organic compounds such as acetate as the carbon source and sulfate/H_2_ as the terminal electron acceptor. Hence, it is postulated that acetate generated by acetogenic bacteria indirectly enhances iron corrosion *via* stimulation of EMIC-inducing SRB (Mand et al., 2014). It is important to mention that apart from SRB, the biofilm of nitrogen-reducing bacteria (NRB) also caused severe corrosion of C1018 carbon steel (Xu et al., 2013). In case of NRB, extracellular electrons generated from iron oxidation are transported into the cytoplasm for nitrate reduction leading to MIC. In a 1-week lab test with *Bacillus licheniformis* (NRB), Xu et al. (2013) reported increased corrosion rates with 14.5 μM pit depth and 0.89 mg/cm^2^ normalized weight loss of C1018 carbon steel. This work substantiates the need to investigate the MIC mechanism of other non-SRB bacteria and study synergistic relation between the two groups of bacteria (NRB and SRB).

## Inhibition and Control of Sulfate-Reducing Bacteria Biofilm Induced Microbiologically Influenced Corrosion

### Chemical Methods of Biofilm/Microbiologically Influenced Corrosion Control and Inhibition

Biofilms protect sessile bacteria from biocide attacks because dense biofilms with sessile cells glued together by EPSs increase mass transfer resistances (Chen et al., 1993; Stoodley et al., 1998). In general, an enormous amount of biocide and chemicals are required to treat such dense biofilm. As a matter of concern, there has been an increase in restrictions on environmental regulations and safety concerns; it is therefore highly desirable to make more effective use of biocides at a low concentration (Gaylarde et al., 1999). The use of high biocide concentrations can be circumvented by adding biocide enhancers such as ethylenediamine disuccinate (EDDS). Such enhancers increase the efficacy of biocides used in treatment for biofilm (Fu, 2013; Ashraf et al., 2014).

The biocide, glutaraldehyde, is well known for its unique capability to suppress or lengthen the growth of SRB cells rather than destroy the cells (Wen et al., 2010). In addition, glutaraldehyde can cross-link proteins and, therefore, can also be used as a common fixative (contains 2.5 wt% glutaraldehyde) (Hayat, 1981). However, due to the toxicity of glutaraldehyde to aquatic life, its use is limited (Leung, 2001). When the biodegradable chelator EDDS is supplemented with a low amount of glutaraldehyde, it enhances the penetration of glutaraldehyde to SRB biofilm (Kumari et al., 2020). Wen et al. (2009) observed that sessile SRB was eradicated from the surface of carbon steel with 30 ppm of glutaraldehyde and 2,000 ppm EDDS, while 30 ppm of glutaraldehyde alone was not effective against the sessile cells. Another study found that a combination of methanol (15%), glutaraldehyde (50 ppm), and EDDS (1,000 ppm) completely removed sessile cells from the biofilm. In contrast, without EDDS [only methanol (15%) and glutaraldehyde (50 ppm)], biofilm was still present (Wen et al., 2012). These studies indicate that chelating agents such as EDDS can be used to increase the biocidal efficacy and reduce the environmental impact of biocides such as glutaraldehyde.

Another study (Xu et al., 2012) reported improved biofilm mitigation when D-methionine was used as an enhancer with the biocide tetrakis-hydroxymethyl phosphonium sulfate (THPS). The effectiveness of THPS (50 ppm) in dispersing DVH biofilm grown on C1018 carbon steel increased when it was used in combination with D-methionine (100 ppm). However, even a higher concentration of THPS (1,000 ppm w/w) alone was found ineffective in biofilm mitigation (Xu et al., 2012). Similarly, other biocides such as glyceryl trinitrate (GTN) and caprylic acid (CA) are also capable of mitigating DVH biofilm formation by delaminating the intact DNA resulting in the inhibition of duplication of microbes (Li et al., 2016). Li et al. (2016) observed that probable sessile cell number count reduced to 10^3^ cells/cm^2^ from 10^6^ cells/cm^2^ when treated with a cocktail of GTN (25 ppm) and CA (0.1%).

Besides, biocides, mannose, and its analogs such as 2-deoxy-D-glucose are also potential biofilm-mitigating agents. Poosarla et al. (2017) observed up to 50% inhibition in SRB biofilm formation when 30 mM mannose was used. Furthermore, up to 90% of biofilm was inhibited at higher concentrations of mannose (100–500 mM). Since mannose can be a possible alternative carbon source, an analog of mannose, 2-deoxy-D-glucose (500 mM), was used, resulting in 94% biofilm inhibition. In contrast, 60% inhibition was recorded with 500 mM of methyl α-D-mannopyranoside (ADM) and methyl α-D-glucopyranoside (ADG). Interestingly, ADM and ADG only inhibit biofilm formation without compromising cell growth.

### Biological Methods of Biofilm/Microbiologically Influenced Corrosion Control and Inhibition

Biofilm formation on carbon steel imposes many problems to the petroleum industry like pipe clogging, biofouling, and biocorrosion. Biocorrosion contributes approximately 40% of corrosion of petroleum pipelines. Petroleum industries utilize chemical biocides such as chloride, glutaraldehyde, and quaternary ammonium salts to inhibit SRB biofilm. However, chemical biocides are expensive and toxic to humans and aquatic life due to their persistence in the environment (Summer et al., 2011). The use of antimicrobial substances produced by microorganisms and other plant extracts may be an alternative method to control SRB biofilm growth and MIC.

Many species from the genus *Alcaligenes* possess antagonistic activity, which inhibits the growth of many bacteria (Austin, 1989; Abd Sharad et al., 2016). Abd Sharad et al. (2016) tested *Alcaligenes faecalis* for its ability to inhibit SRB biofilm. Crude ethyl acetate extract (0.2–12.8 mg/ml) of *A. faecalis* showed immediate inhibition against SRB. Similarly, Wood et al. (2018) reported that supernatant of *P. aeruginosa* could disperse more than 98% of the biofilm. They investigated *P. aeruginosa* mutants, defective in rhamnolipids production, to determine the biochemical basis of SRB biofilm dispersal and concluded that rhamnolipids play a role in the dispersal of SRB biofilm (Wood et al., 2018). In addition, antimicrobial substances (AMS) produced by *Streptomyces lunalinharesii* strain 235 have shown to be effective against *D. alaskensis* NCIMB 13491 biofilm on carbon steel surfaces (Pacheco da Rosa et al., 2013; Rosa et al., 2016). The ability of *D. alaskensis* to form biofilm was sixfold less in the carbon steel coupons treated with AMS (0.05 g protein/ml) than the non-treated ones (Rosa et al., 2016). However, AMS did not affect biofilm stability when biofilm was already present on the coupon surface, indicating the need to pretreat steel coupons with AMS before inoculation with *D. alaskensis*. The corrosion rates of AMS (0.05 g protein/ml)-treated steel also decreased to 0.27 mm/year compared to the untreated control (0.38 mm/year). Overall, it was observed that the use of AMS produced by *S. lunalinharesii* strain 235 to control biofilm formation would be immensely useful in preventing MIC from SRB (Rosa et al., 2016). Another study carried out by Korenblum et al. (2013) elucidated the antimicrobial effect of lemongrass oil (LEO) on SRB. Essential oils are an amalgamation of lipophilic and volatile substances that tend to have the potential for antimicrobial activity. Unlike other natural antimicrobial compounds, essential oils inhibit biofilm growth at the same concentration in planktonic and sessile cells. Therefore, when using essential oil as an antimicrobial agent, the sessile cells cannot protect the organism while forming biofilms. The main component of lemongrass oil is “citral,” which shows antimicrobial activity against several bacteria like *Bacillus cereus*, *S. aureus*, and *E. coli*. LEO treatment causes cell lysis and disruption of the cell wall at different layers of polysaccharides, fatty acids, and phospholipids that ultimately result in cell death. LEO and citral can be used in formulations like synthetic biocides in petroleum industries to prevent SRB-induced metal corrosion and reservoir souring (Korenblum et al., 2013).

The use of other naturally occurring compounds such as plant extracts show potential inhibitory effects against yeast, fungi, and bacteria and can be used to treat MIC (Heisey and Gorham, 1992; Bhola et al., 2014). For example, Neem (*Azadirachta indica*) extract (4%) was reported to reduce the SRB-induced biocorrosion of pipeline steel (API 5L X80) coupons by approximately 50%. Neem extract significantly reduced the contribution of SRB in the corrosion process by minimizing SRB cells’ growth rate and decreasing the sulfide productions and sessile cell density (i.e., biofilm development) (Bhola et al., 2014). In addition, Neem extracts can form adsorbed intermediates on the metal surface (organo-metallic complexes), thereby providing a protective shield to the metal surface (Okafor et al., 2010). Reduction in SRB-induced corrosion by 50% is a significant achievement, as it will reduce biocide use in petroleum industries.

In addition to AMS and plant extracts, new biocides based on bacteriolytic phages are being investigated (Summer et al., 2011). Phages can lyse bacterial cells in single and multispecies biofilm (Doolittle et al., 1995; Sillankorva et al., 2010). For example, phage T4 can disrupt *E. coli* biofilm morphology by killing bacterial cells through infection and replication within the bacteria (Lu and Collins, 2007). Summer et al. (2011) isolated SRB-specific phages capable of inhibiting the growth of species from the genera *Desulfovibrio*. The phage used did not wholly inhibit all SRB, and eventually, the growth was back to normal. However, the usefulness of phage cannot be disregarded, as only a single wild-type phage was used. A cocktail of several phages can be used to ensure complete suppression of bacterial growth (Summer et al., 2011). Another useful strategy for disrupting bacterial biofilm is the enzymatic degradation of EPS. Studies have demonstrated that enzymatic degradation of cell-bound EPS polysaccharides can reduce biofilms of several different species of bacteria (Itoh et al., 2005; Ferriol-González and Domingo-Calap, 2020). In addition, phages can be engineered to express biofilm-degrading enzymes (lysins and depolymerases) to disrupt the biofilm structure. The application of such engineered (enzymatic) phage substantially decreased bacterial biofilm cell counts by ∼4.5 orders of magnitude (∼99.997% removal) (Lu and Collins, 2007). Phage-based methods for biofilm disruption would allow for the design of appropriate and specific biofilm control strategies for different environmental and industrial settings. A brief overview of some chemical and biological compounds used in biofilm inhibition and their mode of action is given in Supplementary Table 2.

### Protective Coatings Based on 2D Materials for Preventing Microbiologically Influenced Corrosion

Compared to biocides and cathodic protection approaches, the use of protective coatings remains a viable choice for retarding MIC. Such coatings are typically based on metals or polymers. However, most of these coatings were designed to combat abiotic forms of corrosion (Pierre and Roberge, 2012). Such coatings, especially those based on polymers, suffer from disadvantages when exposed to biological environments. Furthermore, the effectiveness of polymer coatings is significantly reduced due to aggressive microbial activity in biofilms on the underlying polymer-coated metal surfaces (Krishnamurthy et al., 2015). Although polymer coatings serve as an effective barrier for atmospheric corrosion prevention, they adhere weakly to metal surfaces and are prone to rapid microbial degradation under immersion conditions (Roy et al., 2002; Zhang et al., 2008; Guo et al., 2018). Microbial activity can induce pin-hole defects in polymer coatings, which can grow in size, attract aggressive ions onto metallic surfaces, and further accelerate the corrosion process (Mayavan et al., 2013; Krishnamurthy et al., 2015). In addition to being prone to degradation and aggravating corrosion in some cases, the thickness of polymeric coatings also might disrupt the functional properties (e.g., electrical and thermal conductivity) and dimensional tolerances of target metals (Geim and Novoselov, 2007; Krishnamurthy et al., 2015; Chilkoor et al., 2019). Here, we discuss an emerging class of protective coatings based on 2D materials, including graphene and hexagonal boron nitride. Atomic layers of 2D materials can grow as conformal layers on metallic surfaces using thin film deposition techniques, including chemical vapor deposition (CVD). These 2D materials are mechanically robust, hydrophobic, chemically inert, thermally, and electrically conductive and can form an impermeable barrier (Balandin et al., 2008; Bunch et al., 2008; Krishnamurthy et al., 2015). Due to these characteristics, these 2D materials can be used as an ultrathin protective coating for preventing both abiotic and biotic forms of corrosion. For instance, Krishnamurthy et al. (2013) found that three to four atomic layers of graphene offer long-term resistance (∼40 days) to bimetallic corrosion of Ni, especially under microbial conditions. Their results indicated that graphene coatings appear to combat MIC by (i) preventing access of microbes to the Ni surface, (ii) forming a protective barrier to minimize charge (Ni^2+^) transport into the solution, and (iii) protecting the Ni surface from microbial byproducts (e.g., H^+^) that enhance Ni dissolution. In another study, two commercial polymeric coatings, Parylene-C (PA) and Polyurethane (PU), were compared to ultrathin graphene skin (Gr) coating toward protection against MIC (Krishnamurthy et al., 2015). It was found that MIC on Gr-coated Ni was an order of magnitude lower than PA and PU coated. Gr coating showed ∼10- and ∼100-fold improvement in MIC resistance compared to PA and PU coated. In a more recent study, Chilkoor et al. (2021) found that multilayered Gr coating on Cu and Ni is more suited for MIC prevention when compared to single-layer Gr. Multilayered graphene (MLG) on Cu restricted the MIC by 10- and 1.4-fold compared to single-layer Gr–Cu and bare Cu, respectively. Single-layered graphene on Cu worsened increased MIC by fivefold compared to bare Cu due to the presence of point defects in single-layer graphene, which can accelerate the electrochemical corrosion process (Mayavan et al., 2013). In one another study, atomically thin layers (∼4) of hexagonal boron nitride (hBN) were tested as a protective coating to inhibit MIC of the underlying copper (Cu) surfaces (Chilkoor et al., 2020). The results showed that hBN-coated Cu had six- to sevenfold lower corrosion than bare Cu in abiotic (sulfuric acid and sodium sulfide) and biotic (DAG-G20 bacteria medium) environments. Overall, these studies show that few atomic layers of 2D materials show promise for MIC prevention. However, the use of 2D materials for MIC prevention is a relatively new concept, and more studies are needed to understand nanoscale surface properties, genes activated/deactivated during cell attachment on 2D materials, biofilm growth, and biogenic sulfide attack.

## Conclusion

Biofilm formed by SRB induces changes on the metal surface that accelerate the anodic/cathodic process, controlling corrosion rates. The formation of biofilm is tightly regulated by several genes and the prevailing stress conditions. This review summarized the molecular mechanisms of biofilm formation by SRB and its implications in MIC. The critical genes, proteins, and quorum sensing (QS) molecules are highlighted, which were reported to be involved in biofilm formation irrespective of SRB type. Besides, we provided a theoretical understanding of two mechanisms of MIC by SRB: cathodic depolarization theory (CDP) and biocatalytic sulfate reduction theory (BCSR). CDP accelerates corrosion rates by increased consumption of cathodic hydrogen, whereas BCSR accelerates corrosion rates by increased sulfate reduction rate at the cathode. CDP and BCSR use the extracellular electron transfer (EET) pathway of SRB, as the cathodic reaction is stimulated through the EET pathway in SRB. The genes involved in EET are differentially expressed in SRB biofilm, resulting in enhanced MIC of metal surfaces. To understand MIC, it is essential to recognize the genetic level changes in SRB during biofilm formation. At the genetic level, the expression pattern of hydrogenase (Fe and NiFe) and heme-containing cytochromes regulated corrosion rates (Caffrey et al., 2008). The knockout of genes encoding for hydrogenases and heme-containing cytochromes lowered corrosion rates of iron. In addition, the presence of biofilm significantly increased MIC rates; the inhibition of quorum sensing system would prevent biofilm formation and lower down corrosion rates. However, the genetic level correlation between SRB biofilm and MIC is still lacking. In SRB biofilm, significantly upexpressed genes belong to three functional categories: (1) energy production and conversion, (2) signal transduction, and (3) cell motility. Future studies should focus on understanding the role of specific genes and their interacting partners during MIC. This strategy in combination with a greater understanding of QS system would enable us to design more efficient strategies for corrosion mitigation. In addition, we have reviewed various biological and chemical methods that have been used to disperse biofilms formed by SRB. Biological methods are preferred over chemical methods, as chemical biocides are toxic to humans and aquatic life due to their persistence in the environment. In addition to biocides (chemical or biological), studies on the use of various commercial coatings are gaining increased attention. The use of 2D materials such as graphene as the coating has shown exciting results in inhibiting SRB biofilm formation and MIC. Future studies would require understanding the changes in genetic and molecular mechanisms in SRB during biofilm formation on coated surfaces and designing strategies to overcome biofilm formation and MIC.

## Author Contributions

AT, PT, PS, and SR did literature search and prepared a draft. ViG wrote Section “Role of Quorum Sensing in Sulfate-Reducing Bacteria Biofilm Formation” and reviewed the manuscript. RNS, VeG, EG, and BJ reviewed the manuscript. RKS participated in manuscript design, preparation, and review as well as submissions. All authors contributed to the article and approved the submitted version.

## Conflict of Interest

The authors declare that the research was conducted in the absence of any commercial or financial relationships that could be construed as a potential conflict of interest.

## Publisher’s Note

All claims expressed in this article are solely those of the authors and do not necessarily represent those of their affiliated organizations, or those of the publisher, the editors and the reviewers. Any product that may be evaluated in this article, or claim that may be made by its manufacturer, is not guaranteed or endorsed by the publisher.
