# Supplementary material for: Gene Sets and Mechanisms of Sulfate-Reducing Bacteria Biofilm Formation and Quorum Sensing With Impact on Corrosion
Source: Front Microbiol. 2021 Oct 29;12:754140. doi: 10.3389/fmicb.2021.754140 (PMC8586430; doi:10.3389/fmicb.2021.754140)
Supplement: Supplementary file 2 [file Table_2.DOCX]

Table 2. Biofilm inhibiting chemical and biological compounds along, their mode of action and inhibitory studies.

| Compounds | Mode of Action of Inhibiting biofilm formation | Inhibitory Studies on SRB growth | Negative impact on environment | References |
| --- | --- | --- | --- | --- |
| Chemical Compounds | | | | |
| Glutaraldehyde | Suppressing or lengthening of the cell growth.  Crosslinking of proteins. | Retardation in growth to 143 hours using 50 ppm of glutaraldehyde. | High | (Hayat 1981, Wen,  Zhao et al. 2010) |
| Ethylenediaminedisuccinate (EDDS) | Chelation of cations key in biofilm matrices.  Enhancement in the penetration of glutaraldehyde to SRB biofilm | 2000 ppm of EDDS supplemented with 30 ppm of glutaraldehyde retarded growth to 212 hours | Moderate | (Wen, Zhao et al. 2009, Sharma,  Liu et al. 2018, Kumari,  Rathore et al. 2020) |
| Tetrakis hydroxymethyl phosphonium sulfate (THPS) | Denaturation of proteins  Damages membranes,  Interruption of proton flux and the ADP-ATP energy cycle (Inhibits sulfate reduction pathway) | 1000ppm (w/w) of THPS was found to be ineffective when used solely as a biocide for biofilm mitigation | Moderate | (Xu, Li et al. 2012,  Xue and Voordouw 2015) |
| D-methionine | Biocide enhancer | Higher mitigation of biofilm observed when 50 ppm of THPS was combined with 100 ppm of D-methionine (100ppm) | Low | (Xu, Li et al. 2012) |
| Glyceryl trinitrate (GTN) | Nitride oxide donor and a bioregulatory agent responsible for genomic alterations and delaminating the intact DNA, (inhibits the duplication of microbes) | 3 log reduction of the sessile cell count was achieved using 25 ppm (w/w) of GTN | Low | (Li, Zhang et al. 2016) |
| Caprylic acid (CA) | Acts as antimicrobial agents. | 3 log reductions of the sessile cell count were achieved using 0.1 % (1000 ppm) CA | Low | (Li, Zhang et al. 2016) |
| Biological Compounds | | | | |
| Lemongrass oil | Cell wall disruption due to cellular membrane permeabilization, reduced membrane potential, and the collapse of the proton ion pump | One log reduction of SRB cells | Low | (Korenblum,  Regina de Vasconcelos  Goulart et al. 2013) |
| AMS produced by *Streptomyces lunalinharesii* | Cell death by permeabilization of cell wall | Biofilm formation by SRB is six-fold smaller | Low | (Rosa, Tibúrcio et al. 2016) |
| Azadirachtin | Limits cells metabolic processes to propagate them into the decline phase | 50% reduction in SRB induced corrosion | Low | (Bhola, Alabbas et al. 2014) |
| D-glucosamine | Hydrolyzes β-1,6-N-acetyl-Dglucosamine, a crucial adhesin needed for biofilm formation | Reduced bacterial biofilm cell counts by ~4.5 orders of magnitude (~99.997% removal) | Low | (Lu and Collins 2007) |

**References**

Bhola, S. M., et al. (2014). "Neem extract as an inhibitor for biocorrosion influenced by sulfate reducing bacteria: A preliminary investigation." Engineering Failure Analysis **36**: 92-103.

Hayat, M. A. (1981). Principles and techniques of electron microscopy. Biological applications, Edward Arnold.

Korenblum, E., et al. (2013). "Antimicrobial action and anti-corrosion effect against sulfate reducing bacteria by lemongrass (Cymbopogon citratus) essential oil and its major component, the citral." AMB Express **3**(1): 44.

Kumari, N., et al. (2020). "Bacterial Biofilms and Ethylenediamine-N, N’-disuccinic acid (EDDS) as Potential Biofilm Inhibitory compound." Proceedings of the Pakistan Academy of Sciences: B. Life and Environmental Sciences **57**(1): 85-92.

Li, Y., et al. (2016). "Glyceryl trinitrate and caprylic acid for the mitigation of the Desulfovibrio vulgaris biofilm on C1018 carbon steel." World Journal of Microbiology and Biotechnology **32**(2): 23.

Lu, T. K. and J. J. Collins (2007). "Dispersing biofilms with engineered enzymatic bacteriophage." Proceedings of the National Academy of Sciences **104**(27): 11197.

Rosa, J. P. d., et al. (2016). "Streptomyces lunalinharesii 235 prevents the formation of a sulfate-reducing bacterial biofilm." Brazilian journal of microbiology : [publication of the Brazilian Society for Microbiology] **47**(3): 603-609.

Sharma, M., et al. (2018). "Effect of selected biocides on microbiologically influenced corrosion caused by Desulfovibrio ferrophilus IS5." Scientific reports **8**(1): 1-12.

Wen, J., et al. (2010). "Chelators enhanced biocide inhibition of planktonic sulfate-reducing bacterial growth." World Journal of Microbiology and Biotechnology **26**(6): 1053-1057.

Wen, J., et al. (2009). "A green biocide enhancer for the treatment of sulfate-reducing bacteria (SRB) biofilms on carbon steel surfaces using glutaraldehyde." International Biodeterioration & Biodegradation **63**(8): 1102-1106.

Xu, D., et al. (2012). "A synergistic D-tyrosine and tetrakis hydroxymethyl phosphonium sulfate biocide combination for the mitigation of an SRB biofilm." World Journal of Microbiology and Biotechnology **28**(10): 3067-3074.

Xue, Y. and G. Voordouw (2015). "Control of microbial sulfide production with biocides and nitrate in oil reservoir simulating bioreactors." Frontiers in Microbiology **6**: 1387.
